# Prioritised identification of structural classes of natural products from higher plants in the expedition of antimalarial drug discovery

**DOI:** 10.1007/s13659-023-00402-2

**Published:** 2023-10-12

**Authors:** Phanankosi Moyo, Luke Invernizzi, Sephora M. Mianda, Wiehan Rudolph, Andrew W. Andayi, Mingxun Wang, Neil R. Crouch, Vinesh J. Maharaj

**Affiliations:** 1 Department of Chemistry, Faculty of Natural and Agricultural Sciences, Biodiscovery Center, University of Pretoria, Private Bag X 20, Hatfield, Pretoria, 0028 South Africa; 2 Department of Physical and Biological Sciences, Murang’a University of Technology Murang’a, Murang’a, Kenya; 3 Computer Science and Engineering, University of California Riverside, 900 University Ave, Riverside, CA 92521 USA; 4 Biodiversity Research and Monitoring Directorate, South African National Biodiversity Institute, Berea Road, P.O. Box 52099, Durban, 4007 South Africa; 5 School of Chemistry and Physics, University of KwaZulu-Natal, Durban, 4041 South Africa

**Keywords:** Natural products, Compound classes, Phytochemicals, *Plasmodium falciparum*, Antiplasmodial drug resistance, Malaria, Drug development

## Abstract

The emergence and spread of drug-recalcitrant *Plasmodium falciparum* parasites threaten to reverse the gains made in the fight against malaria. Urgent measures need to be taken to curb this impending challenge. The higher plant-derived sesquiterpene, quinoline alkaloids, and naphthoquinone natural product classes of compounds have previously served as phenomenal chemical scaffolds from which integral antimalarial drugs were developed. Historical successes serve as an inspiration for the continued investigation of plant-derived natural products compounds in search of novel molecular templates from which new antimalarial drugs could be developed. The aim of this study was to identify potential chemical scaffolds for malaria drug discovery following analysis of historical data on phytochemicals screened in vitro against *P. falciparum*. To identify these novel scaffolds, we queried an in-house manually curated database of plant-derived natural product compounds and their in vitro biological data. Natural products were assigned to different structural classes using NPClassifier. To identify the most promising chemical scaffolds, we then correlated natural compound class with bioactivity and other data, namely (i) potency, (ii) resistance index, (iii) selectivity index and (iv) physicochemical properties. We used an unbiased scoring system to rank the different natural product classes based on the assessment of their bioactivity data. From this analysis we identified the top-ranked natural product pathway as the alkaloids. The top three ranked super classes identified were (i) pseudoalkaloids, (ii) naphthalenes and (iii) tyrosine alkaloids and the top five ranked classes (i) quassinoids (of super class triterpenoids), (ii) steroidal alkaloids (of super class pseudoalkaloids) (iii) cycloeudesmane sesquiterpenoids (of super class triterpenoids) (iv) isoquinoline alkaloids (of super class tyrosine alkaloids) and (v) naphthoquinones (of super class naphthalenes). Launched chemical space of these identified classes of compounds was, by and large, distinct from that of ‘legacy’ antimalarial drugs. Our study was able to identify chemical scaffolds with acceptable biological properties that are structurally different from current and previously used antimalarial drugs. These molecules have the potential to be developed into new antimalarial drugs.

## Introduction

Despite a plethora of resolute regional and global concerted efforts to curb malaria, this infectious disease continues to be a considerable health burden particularly to the populace in low-income countries of Africa and Asia [1]. In 2021 there were ca. 245 million reported clinical cases of malaria globally. The World Health Organisation (WHO) African Region carried the highest burden of this disease with ca. 234 million cases accounting for ca. 95% of global clinical malaria cases [1]. Significantly, four Sub-Saharan African countries accounted for 50% of the global clinical malaria burden in 2021 [1]. Despite reporting a low number of clinical malaria cases over the years, the South-East Asia region is notorious for being the epicenter of antimalarial drug-resistance development [2]. This comes as earlier chloroquine and sulphadoxine-pyrimethamine drug-resistant *P. falciparum* strains first emerged from this territory before spreading to the rest of the world [2]. Unfortunately, this phenomenon is recurring in South-East Asia as evidenced by the emergence of *P. falciparum* strains resistant to the current first-line WHO recommended antimalarial therapeutics, the artemisinin-based combination therapy (ACT) [2, 3]. There is genuine solicitude that the inadvertent spread of these ACT-resistant *P. falciparum* strains to Africa will result in a catastrophic outbreak of the disease, undermining momentous efforts to eliminate and eradicate malaria [2]. In this context, there is an urgent need to discover and develop new antimalarial drugs to circumvent this imminent global health threat. One source worth exploring as a starting point in this regard is higher plant-derived natural products.

**Fig. 1:**
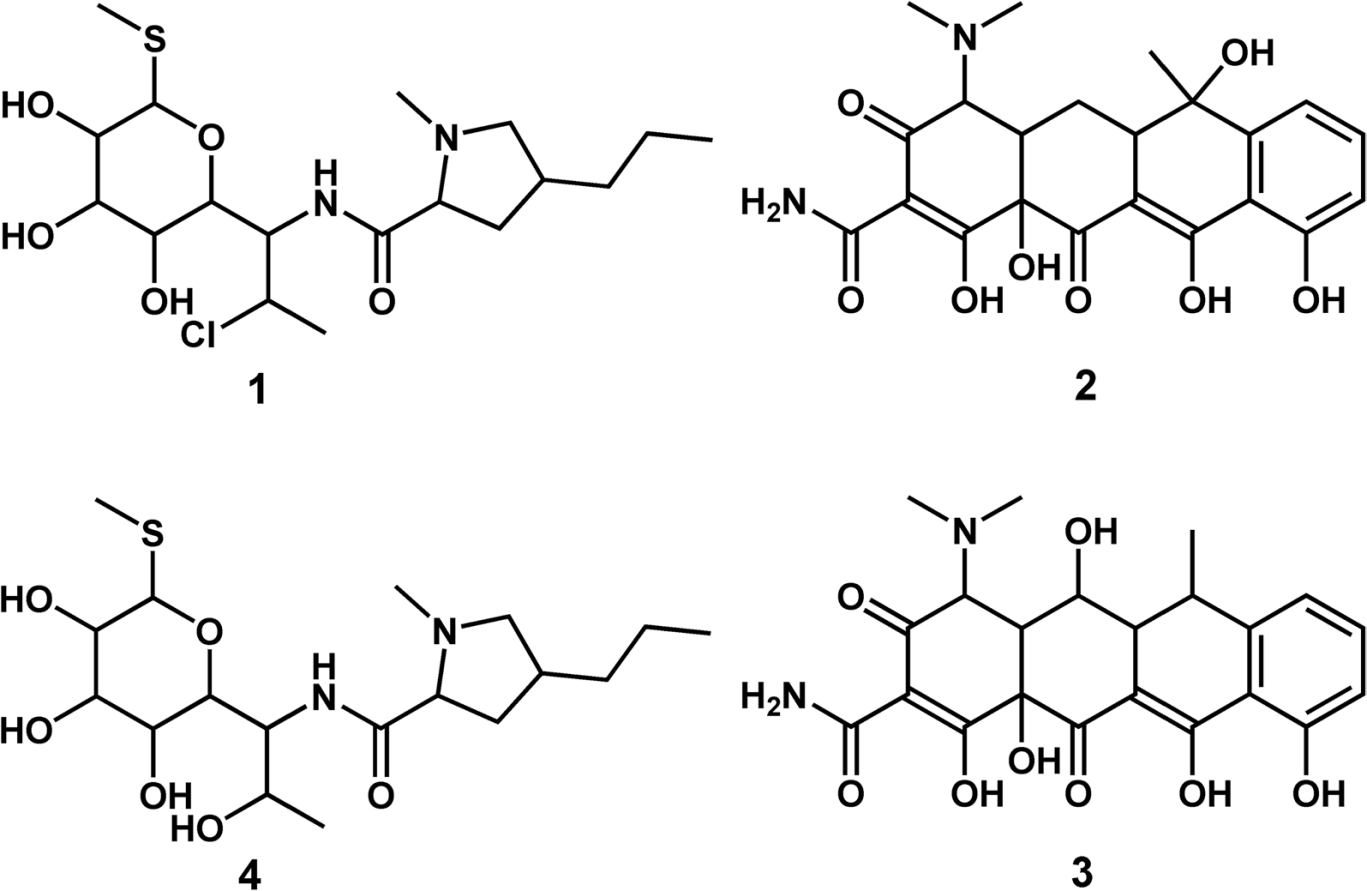
2D illustration of bacterial (*Streptomyces*) derived antimalarial drugs. **1** and **4** are aminoglycosides, and **2** and **3** are tetracyclines

**Fig. 2:**
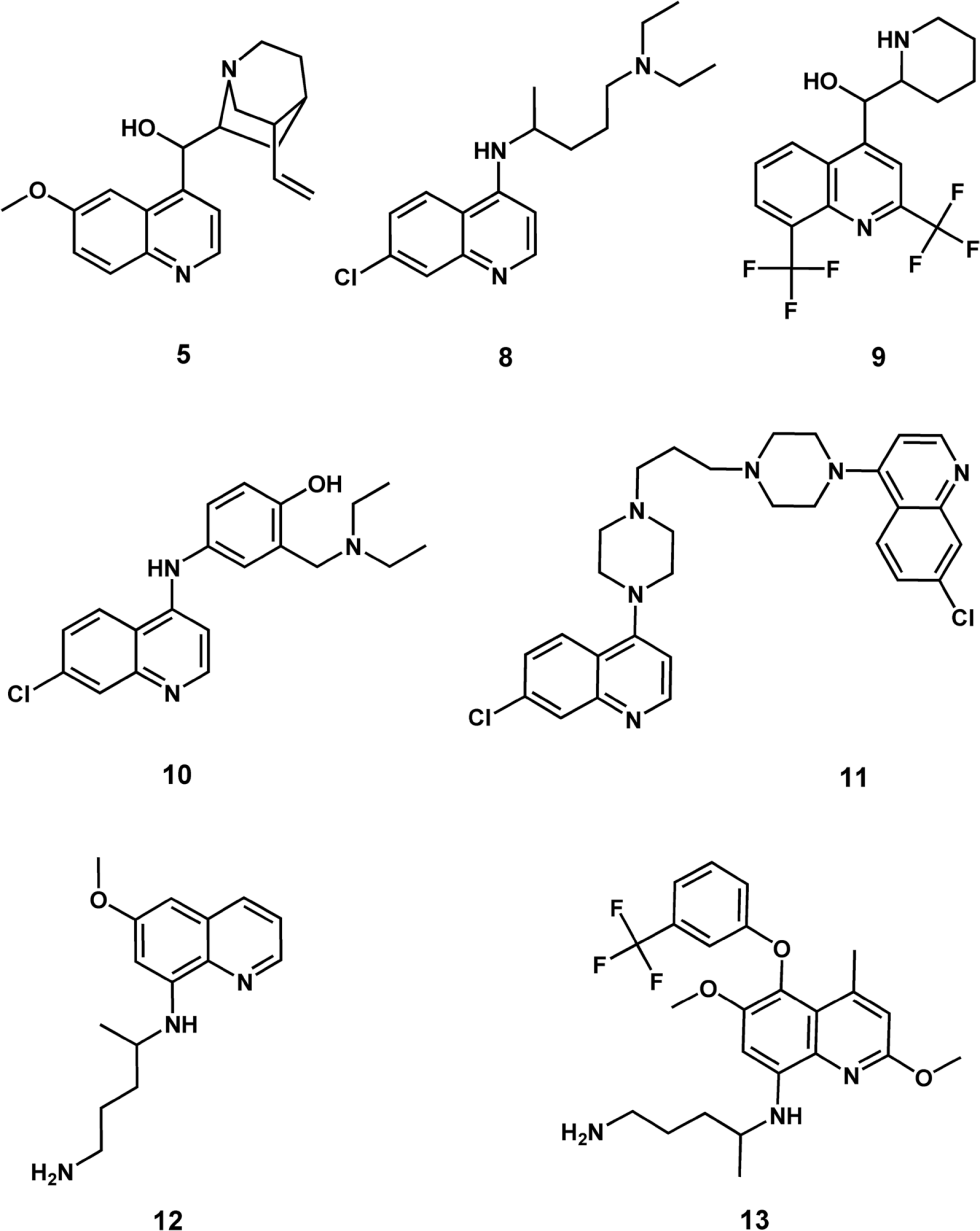
2D illustration of plant-derived antimalarial drug compounds quinine (**5**) and its derivatives the 4-aminoquinolines **8**–**11** and 8-aminoquinolines **12** and **13**

**Fig. 3:**
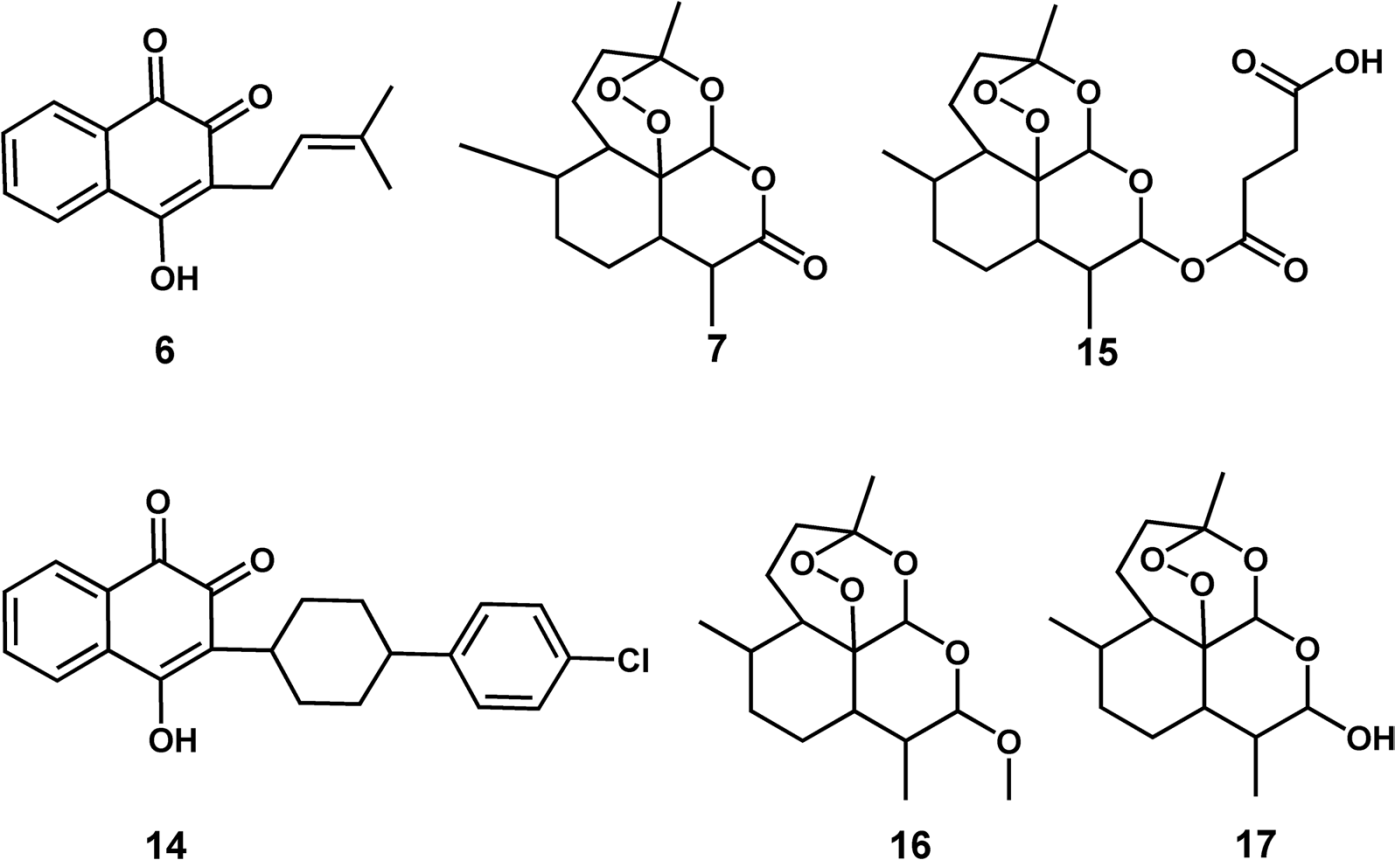
2D illustration of plant-derived antimalarial drug compounds lapachol (**6**) and artemisinin (**7**) and their derivatives **14** and **15**–**17**, respectively

Different classes of both microbial and plant-derived natural products have historically proven to be an indispensable source of lead compounds for the development of the antimalarial arsenal [4]. Three derivatives of microbial-produced natural product compounds remain clinically useful for malaria control. These are clindamycin (**1**) (Fig. 1), tetracycline (**2**) (Fig. 2), and doxycycline (**3**) (Fig. 3) [5, 6]. Clindamycin is a lincosamide antibiotic [7]. It is derived from the *Streptomyces*-produced natural product compound lincomycin (**4**) which belongs to the aminoglycoside class of natural products (Fig. 1) [7]. Tetracycline and doxycycline are 1st and 2nd generation semi-synthetic derivatives, respectively, belonging to the tetracycline class of compounds [8]. Tetracyclines were originally isolated from filamentous bacteria of the genus *Streptomyces* [8]. Each one of these three drugs, **1**–**3**, are co-administered with either artesunate or quinine and used as a second line treatment regimen for recurrent *P. falciparum* malaria. Furthermore, a combination of clindamycin and quinine is strongly recommended by the WHO for treating uncomplicated *P. falciparum* malaria in the first trimester of pregnancy [6].

Critical in the battle against malaria has been the contribution of plant-derived natural products. These phytochemicals are quinine (**5**), lapachol (**6**), and artemisinin (**7**) which belong to the quinoline alkaloid, naphthoquinone and sesquiterpene lactone classes of natural products, respectively (Figs. 2, 3) [4]. Quinine served as a template from which its derivatives, the aminoquinolines, including chloroquine (**8**), mefloquine (**9**), amodiaquine (**10**), piperaquine (**11**), and primaquine (**12**) were developed (Fig. 2) [4]. The most recent quinine derivative that has come to the fore is tafenoquine (**13**) (Fig. 2) [9]. For decades, chloroquine, an affordable and highly efficacious drug, was successfully used as the first line treatment drug for malaria. Mefloquine, amodiaquine and piperaquine are part of the ACT regimen [6]. Primaquine and tafenoquine are both used for preventing relapse of *P. vivax* and *P. ovale* with the former compound additionally used for blocking transmission of sexual *P. falciparum* parasites [6, 10].

Lapachol served as a scaffold which inspired the development of atovaquone (14) [4] which currently, in combination with proguanil, is used as a casual prophylactic for malaria (Fig. 3) [6]. Three prolific semi-synthetic derivatives of artemisinin namely artesunate (15), artemether (16) and dihydroartemisinin (17) are the core components of the ACT regimen (Fig. 3) [6]. By and large, the contribution of natural products, particularly those which are plant-derived, has been profound in the fight against malaria. Against this background, it’s only logical to continue investigating this immense source in search of valuable classes of natural product scaffolds to expedite development of the next generation of antimalarials.

In the past decade there has been an evolution in the natural product-based drug discovery field which allows for the targeted isolation of compounds, a paradigm shift from the classic ‘blind’ resource and time-consuming bioassay-guided approach. This transformation has been spurred on by the introduction of advanced hyphenated analytical techniques such as liquid chromatography coupled with mass spectrometry. Furthermore, there has been an advent of platforms such as GNPS [11] and SIRIUS [12] for high throughput spectral annotation and compound dereplication. Combined, these techniques and tools can facilitate the targeted isolation of structurally related phytochemicals, i.e., a natural “chemical series” of plant-derived analogues (analogous to a chemical series of compounds produced in a synthetic drug discovery program). This “series” of natural analogues can be subjected to biological evaluation with the added advantage of acquiring its structure-activity relationship (SAR) data. The prompt establishment of the SAR for the targeted compound “series” guides precise development of their medicinal chemistry plans, to expedite their development as potential drugs in the discovery pipeline.

For decades, multitudes of phytochemicals of diverse structural classes have been screened in vitro for activity against asexual *P. falciparum* parasites. Given the immense structural variety and number of plant-derived natural products (ca. 133 881 plant-derived compounds reported in the dictionary of natural products by 2019 [13]), there is a need for a rational approach to prioritise classes of compounds for malaria drug discovery projects. This approach has been attempted in a previous study by Egieyeh and co-workers which primarily carried out a chemoinformatic-based analysis of a set of 1040 antiplasmodial natural compounds isolated from different sources including plants, microorganisms, and marine species [14]. Furthermore, this prior study briefly examined the relationship between chemical class and bioactivity. The outcome of the study was a list of specific natural compounds the authors recommended be prioritised for antimalarial drug discovery [14]. In accordance with this previous work, we here analyse an in-house data set of 2400 plant-derived natural compounds to ascertain which structural classes of natural compounds at the pathway, super class and class levels should reasonably be prioritised for antimalarial drug discovery. However, in variance to the study of Egieyeh et al., emphasis is herein placed on prioritising structural classes and not individual compounds. Furthermore, our work has delved more into the potency, additionally considering activity of the compounds against both drug-sensitive (D-S) and drug-resistant (D-R) intra-erythrocytic *P. falciparum* parasites, an aspect not previously undertaken by the earlier workers.

In this current study, we first evaluated the structural classes, namely pathways, super class, and class, based on four parameters, (i) potency, (ii) resistance index (RI), (iii) selectivity index (SI), and (iv) drug-likeness properties. We then used an unbiased scoring mechanism to rank structural classes based on their performance in two (potency and SI) of these four parameters. From the ranked list, the topmost pathway, super class, and class categories were subsequently identified. We envisage that findings from our study will be of value to the malaria drug discovery field and will potentially play a role in hastening the discovery of novel antimalarial chemotypes through target-based isolation.

## Results, discussion and conclusion

### Descriptive analysis: hit rate of natural product compounds in different pathways, super class and class categories

To identify the most quintessential structural class of plant-derived natural compounds for antimalaria drug discovery and development, we examined our previously manually curated database (Moyo et al. submitted). The database consists of a set of 2400 plant-derived natural compounds (representing 1.8% of known phytochemicals [13]) previously evaluated in vitro for their antiplasmodial activity against the intra-erythrocytic asexual *P. falciparum* parasites. These plant-derived compounds were identified and compiled from peer-reviewed literature sources from PubMed published over a course of 58 years between 1964 and 2021. Additional information available on the database included the pharmacological activity of the phytochemicals including their in vitro antiplasmodial potency against intra-erythrocytic asexual *P. falciparum* parasites, and cytotoxicity data.

**Fig. 4:**
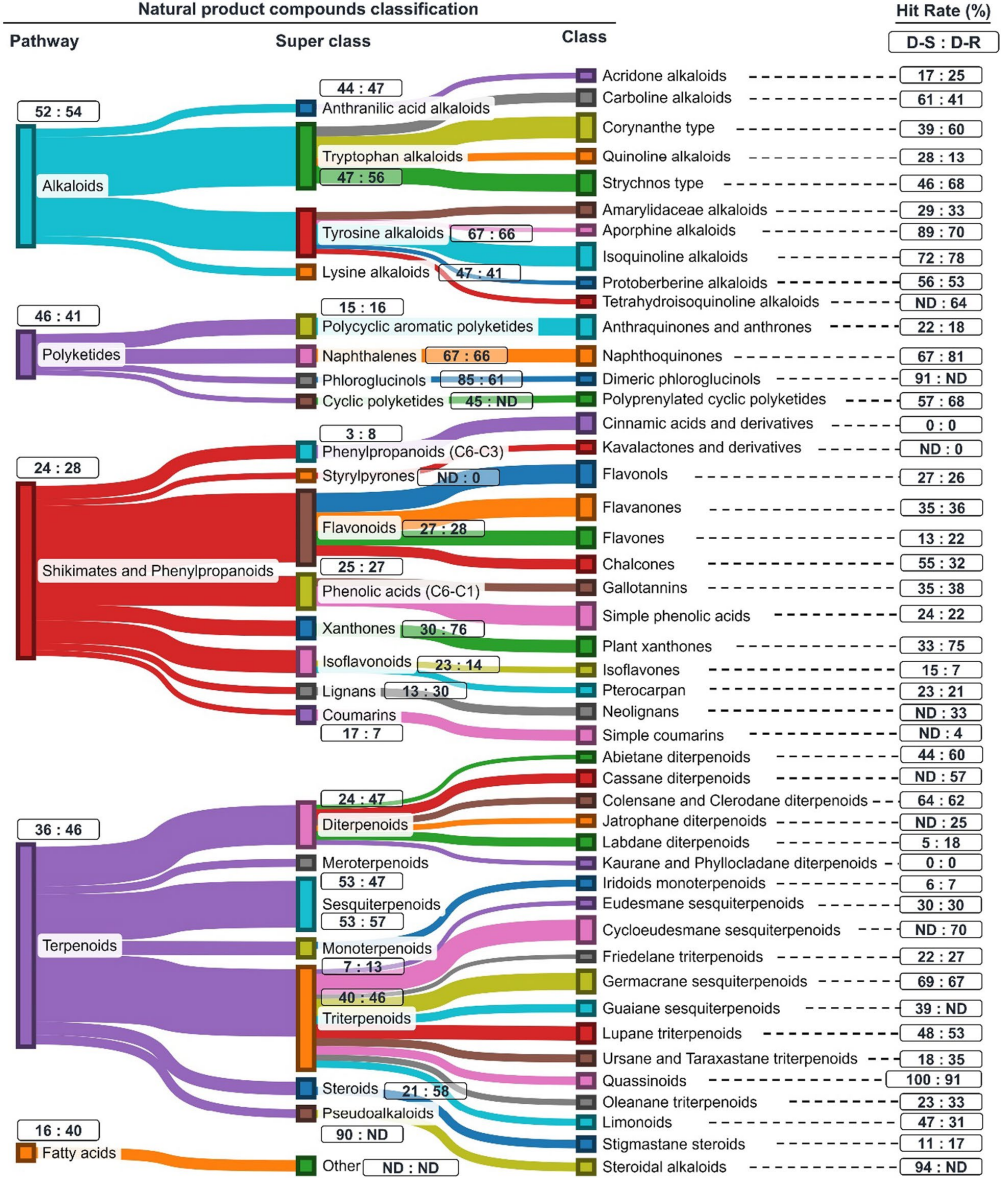
Sankey chart illustration of pathways, super class and class of plant-derived natural compounds examined in this study. The size of nodes is proportional to the relative number of compounds across the different pathways and within the respective super classes and classes. Values in rectangles show hit rate (HR) against drug-sensitive (D-S) and drug-resistant (D-R) intra-erythrocytic asexual *P. falciparum* strains. *ND* not determined: This applies for compound pathways, super class and class of natural product compounds in which < 10 compounds per classification were evaluated for their antiplasmodial activity. The hit rate is the calculated % number of compounds with an IC_50_ ≤ 10 µM for each structural classification. Classification of compounds into pathways, super class and class levels was primarily carried out using the online tool NPClassifier, and secondarily using ClassyFire, both automated online structural classification tools. Sankey chat was created using the online platform SankeyMATIC (https://sankeymatic.com/)

To achieve the objective of our study we first assigned the 2400 natural product compounds to different structural classes, and to reduce subjectivity and ensure consistency used an online chemical ontology classification tool to classify all compounds/structures. Compounds in the database were assigned into three hierarchical classification categories of natural products, namely pathway (highest level), super class (middle level) and class (lowest level). The classification was carried out primarily using the online deep neural network-based structural classification ontology tool NPClassifier (Fig. 4) [15]. However, NPClassifier in some cases apportioned compounds to multiple classes. Hence in such circumstances, another web-based application, ClassyFire [16], was then adopted as an “arbitrator”, resolving this ambiguity, so assigning the compound into one specific class level. NPClassifier has been shown to outperform ClassyFire in accurately assigning secondary metabolites to their correct classes [15], hence its application for the primary classification.

Using NPClassifier, 97% (2349) of the compounds in the database were successfully classified into five different pathways, namely alkaloids, terpenes, polyketides, fatty acids, and shikimates and phenylpropanoids. Two hundred and twelve of the compounds multi-classified by NPClassifier were successfully reclassified at the class level using ClassyFire. Terpenes were the most represented pathway accounting for 40% of plant-derived compounds examined (Fig. 4). They were closely followed by shikimates and phenylpropanoids (30%), while fatty acids (2%) were the least represented. This frequency in the abundance of the pathways is consistent with their reported distribution in plants. This distribution partially matched that of antiplasmodial marine natural products reported elsewhere [17]. At the super class level triterpenoids and flavonoids were the two most prevalent whilst styrylpyrones and cyclic polyketides ranked as the least represented. Only phytochemicals with ≥ 10 compounds at a class level were considered for this study. At this low level of classification, amongst the three most represented classes were isoquinoline alkaloids (of super class tyrosine alkaloids), germacrane sesquiterpenoids (of super class triterpenoids), and corynanthe-type alkaloids (of super class tryptophan alkaloids). Aporphine alkaloids (of super class tyrosine alkaloids) and abietane diterpenoids (of super class diterpenoids) ranked amongst the least represented chemical entities at the class level (Fig. 4).

Having successfully classified the phytochemicals, the next step was to get a preliminary insight into the potency of natural products assigned to the different pathways, super class and class levels. This analysis was carried out by linking different compound classifications to their reported in vitro activity against both D-S and D-R intra-erythrocytic asexual *P. falciparum* parasites. The hit rates (HR) of the compounds per each pathway, super class and class were computed as previously reported by Moyo et al. (in review). The HR is the proportion of active compounds (defined as those with an IC_50_ ≤ 10 µM) relative to the total number of compounds in that structural classification category. Considering the time and exorbitant financial resources invested in drug discovery projects, there is merit in working on structural classes with a high HR, the presumption being that this will ensure drugs are developed faster and more cost-effectively.

Amongst the pathways, alkaloids had the highest proportion of active compounds and so achieved the topmost HR against both D-S (HR = 52%, n (number of compounds) = 281) and D-R (HR = 54%, n = 344) *P. falciparum* strains. Fatty acids had the lowest HR against D-S (HR = 17%, n = 24) while polyketides had the lowest HR against D-R (HR = 28%, n = 117) *P. falciparum* strains (Fig. 4). Interestingly, the fatty acids were more potent against D-R rather than to D-S asexual *P. falciparum* strains (difference of 23 between the HR).

At the super class level, pseudoalkaloids (HR = 90%, n = 20) and naphthalenes (HR = 65%, n = 34) had the highest ratio of active compounds against D-S and D-R *P. falciparum* strains, respectively. Phenylpropanoids (C6–C3) (HR = 3.4%, n = 29) and styrylpyrones (HR = 0%, n = 13) had the lowest ratio of active compounds against D-S and D-R *P. falciparum* parasites, respectively (Fig. 4). Xanthones, steroids, and diterpenoids had a remarkably high proportion of compounds active against D-R relative to D-S *P. falciparum* strains. The differences in the HR for activity against D-S and D-R parasites were 44, 36, and 22.7 for xanthones, steroids, and diterpenoids, respectively.

Quassinoids were revealed to be topmost at the class level, having the highest HR against both D-S (HR = 100%, n = 18) and D-R (HR = 91%, n = 54) intra-erythrocytic asexual *P. falciparum* parasites. Cinnamic acids and its derivatives (of super class phenylpropanoids) (HR = 0%, n = 29 and 23), and kaurane and phyllocladane diterpenoids (of super class diterpenoids) (HR = 0%, n = 10), had the lowest ratio of active compounds against both D-S and D-R asexual *P. falciparum* parasites. Kavalactones and derivatives (of super class styrylpyrones) also had a poor representation of active compounds (HR = 0%, n = 13) against D-R *P. falciparum* parasites. Consistent with its parent super class (xanthones), plant xanthones had a marked proportion of active compounds against D-R compared to D-S parasites (a difference of 44 between the HR). Chalcones (of super class flavonoids), carboline alkaloids (of super class tryptophan alkaloids), and aporphine alkaloids (of super class tyrosine alkaloids) had a higher ratio of active compounds against D-S compared to D-R *P. falciparum* parasites (difference of ca. 19 between HR for all three classes of natural compounds) (Fig. 4).

In general, from this primary analysis we observe that chemical entities within the alkaloid pathway, and its associated super classes and classes have a high HR against both D-S and D-R *P. falciparum* parasites. In contrast, natural product compounds assigned to the shikimates and phenylpropanoids pathway and its related super class and class categories largely demonstrated low HR against the D-S and D-R *P. falciparum* parasite strains.

### Antiplasmodial activity of natural products in different pathways, super classes and classes

Following the preliminary analysis of activity, we further examined the relevant pharmacological properties of compounds assigned to the different structural classification categories. We broadened our evaluation of potency by looking into the proportion of compounds per pathway, super class and class, classified as either highly active (HA) (IC_50_ ≤ 1 µM), moderately active (MA) (10 µM ≥ IC_50_ > 1 µM), or poorly active (PA) (IC_50_ > 10 µM). Furthermore, we investigated the resistance index (RI) and selectivity index (SI) for the reasons outlined above. A pathway, super class and class of priority for malaria drug discovery and development is taken to be one whose majority of compounds are classified as HA, while also having acceptable low RI (≤ 10) and high SI (≥ 10).

From this evaluation, most natural compounds in all five pathways were classified as PA (Table 1). The alkaloids pathway had the greatest number of compounds classified as HA against both D-S (22%, n = 281) and D-R (23%, n = 344) asexual *P. falciparum* parasites (Table 1). Furthermore, many (50%) of the natural products in the alkaloid pathway (n = 232) showed a good SI. Fatty acids and shikimates and phenylpropanoids had the fewest number of compounds classified as HA with most of the molecules (ca. 74%) in the latter pathway (n = 209) demonstrating a poor SI. The emergence of alkaloids as the most prolific pathway is consistent with findings from Egieyeh et al. [14].

**Table 1:**
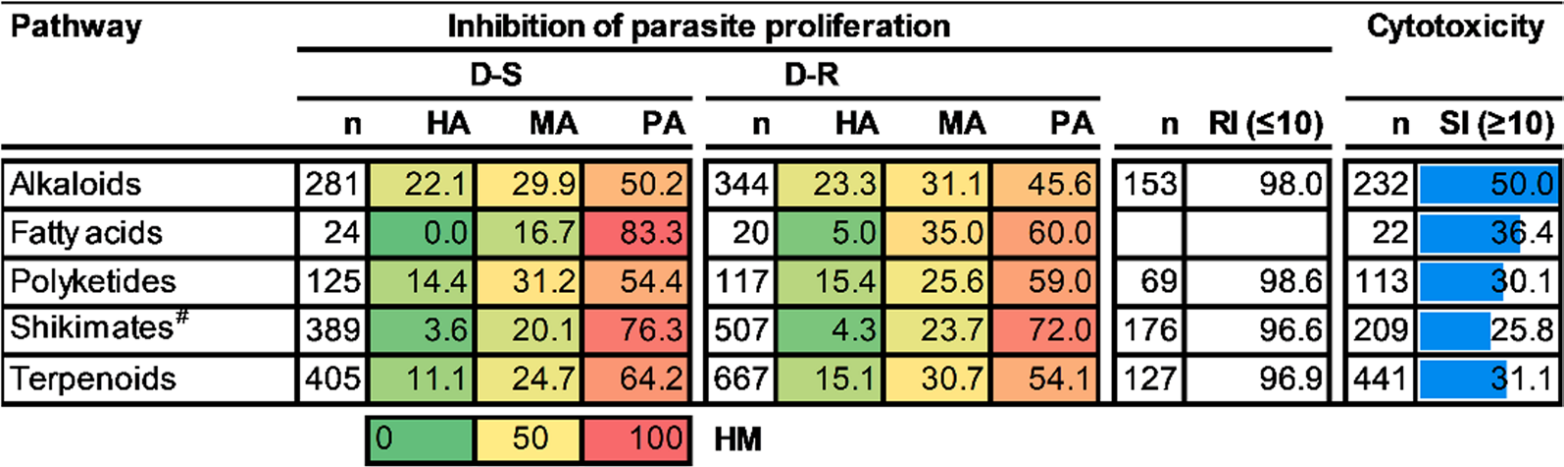
Antiplasmodial activity and cytotoxicity of higher plant-derived natural compound pathways

n = number of compounds; HA, MA and PA values are expressed in % of total compounds evaluated; RI shows % number of compounds with RI ≤ 10; SI shows % number of compounds with SI ≥ 10. The heat map (HM) ranges from green (lowest value, 0%) to yellow (mid-range value, 50%) to red (highest value, 100%), visually illustrating the proportion of compounds classified as either HA, MA or PA. ^#^Shikimates and Phenylpropanoids

For the super class evaluation, most compounds in the naphthalenes were classified as HA against both D-S (43%, n = 30) and D-R (43%, n = 35) asexual *P. falciparum* parasites. Other super classes to have a considerable proportion (> 25%) of their compounds classified as HA against D-S *P. falciparum* parasites were anthranilic acid alkaloids (n = 18), lysine alkaloids (n = 17), tyrosine alkaloids (n = 83) and pseudoalkaloids (n = 20). Against the D-R parasites, anthranilic acid alkaloids (n = 23), tyrosine alkaloids (n = 134) and tryptophan alkaloids (n = 117) had a marked proportion (> 25%) of their compounds classified as HA. Additionally, the majority of compounds (> 45%) from the anthranilic acid alkaloids (n = 8), lysine alkaloids (n = 14), tyrosine alkaloids (n = 106) and pseudoalkaloids (n = 20) showed acceptable SI (> 10). Extensive historical investigations reflected in our database have shown that most compounds (> 70%) of the super classes flavonoids, isoflavonoids, and phenolic acids (C6-C1) were PA against both D-S and D-R asexual *P. falciparum* parasites. Flavonoids, along with terpenoids, have previously been observed to exhibit generally low antiplasmodial activity [14]. Overall, most compounds showed an acceptable RI (< 10), see Table 2.

**Table 2:**
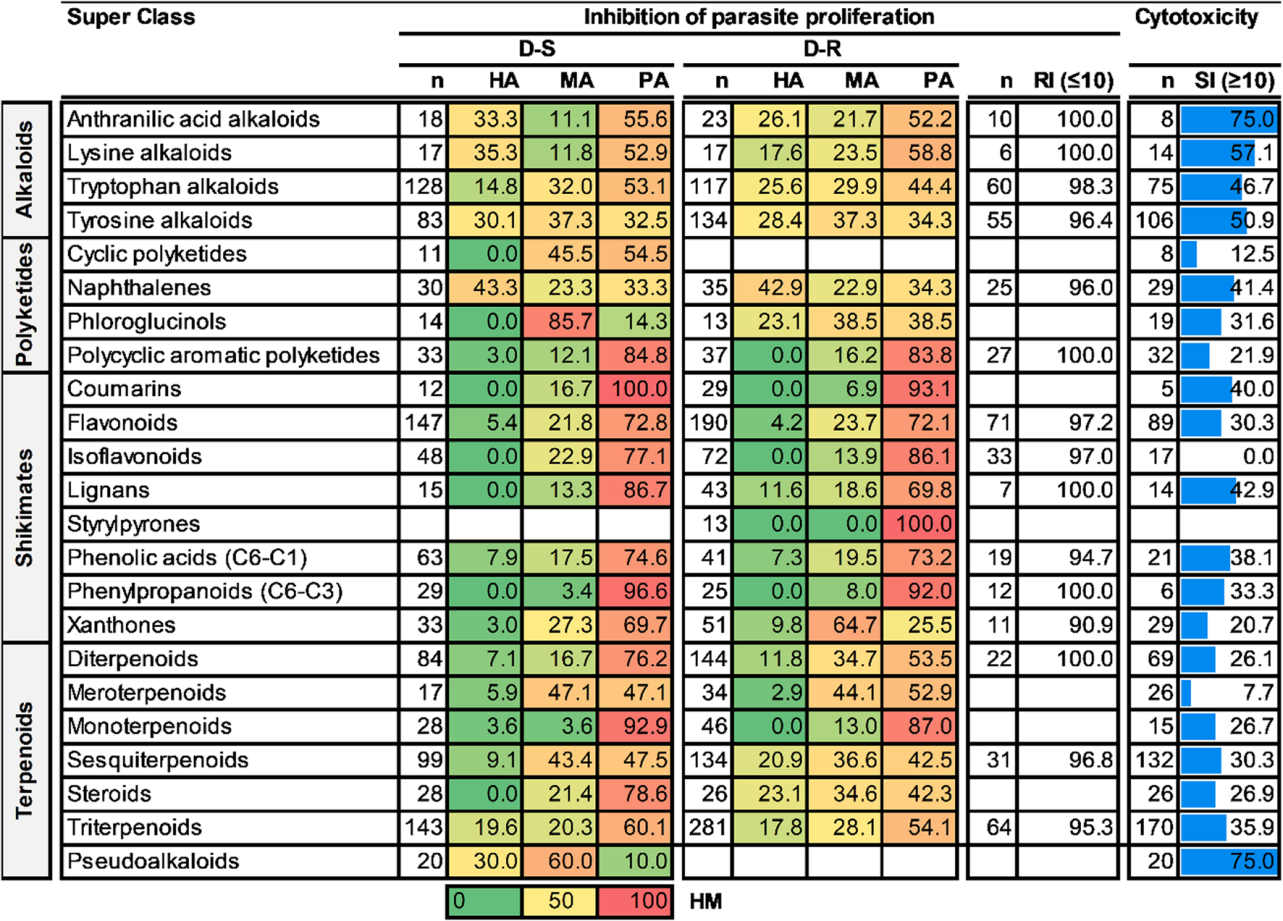
Antiplasmodial activity and cytotoxicity of higher plant-derived super classes of natural compounds

n = number of compounds; HA, MA and PA values are expressed as % of total compounds evaluated; RI shows % of compounds with RI ≤ 10; SI shows % of compounds with SI ≥ 10. The heat map (HM) ranges from green (lowest value, 0%) to yellow (mid-range value, 50%) to red (highest value, 100%), visually illustrating the proportion of compounds classified as either HA, MA or PA. ^#^Shikimates and Phenylpropanoids

At the class level, the potency of quassinoids (of super class triterpenoids) was outstanding in having the majority of the compounds classified as HA against both D-S (77%, n = 18) and D-R (63%, n = 54) *P. falciparum* parasites. This level of potency was followed by naphthoquinones (of super class naphthalenes) whose majority of compounds emerged as HA against both D-S (44%, n = 27) and D-R (57%, n = 26) parasites. Isoquinoline alkaloids (of super class tyrosine alkaloids), steroidal alkaloids (of super class pseudoalkaloids), cycloeudesmane sesquiterpenoids (of super class triterpenoids), cassane diterpenoids (of super class diterpenoids), aporphine alkaloids (of super class tyrosine alkaloids), strychnos type alkaloids (of super class tryptophan alkaloids), and arylnaphthalene and aryltetralin lignans (both of super class triterpenoids) also fared well with > 30% of their compounds classified as HA either against D-S or D-R parasites. A considerable proportion of compounds (ranging from 44 to 84%) from all these mentioned classes also showed acceptable SI. The classes with the lowest proportion of plant-derived natural compounds classified as either HA or MA against *P. falciparum* parasites were kaurane and phyllocladane diterpenoids (of super class diterpenoids), kavalactones and derivatives (of super class styrylpyrones), simple coumarins (of super class coumarins), oleanane triterpenoids (of super class triterpenoids), labdane diterpenoids (of super class diterpenoids), isoflavones (of super class isiflavanoids), iridoids monoterpenoids (of super class monoterpenoids), flavones (of super class flavanoids), cinnamic acids and derivatives (of super class phenylpropanoids), anthraquinones and anthrones (of super class polycyclic and aromatic polyketides) and, interestingly, the quinoline alkaloids (of super class tryptophan alkaloids) (Table 3).

**Table 3:**
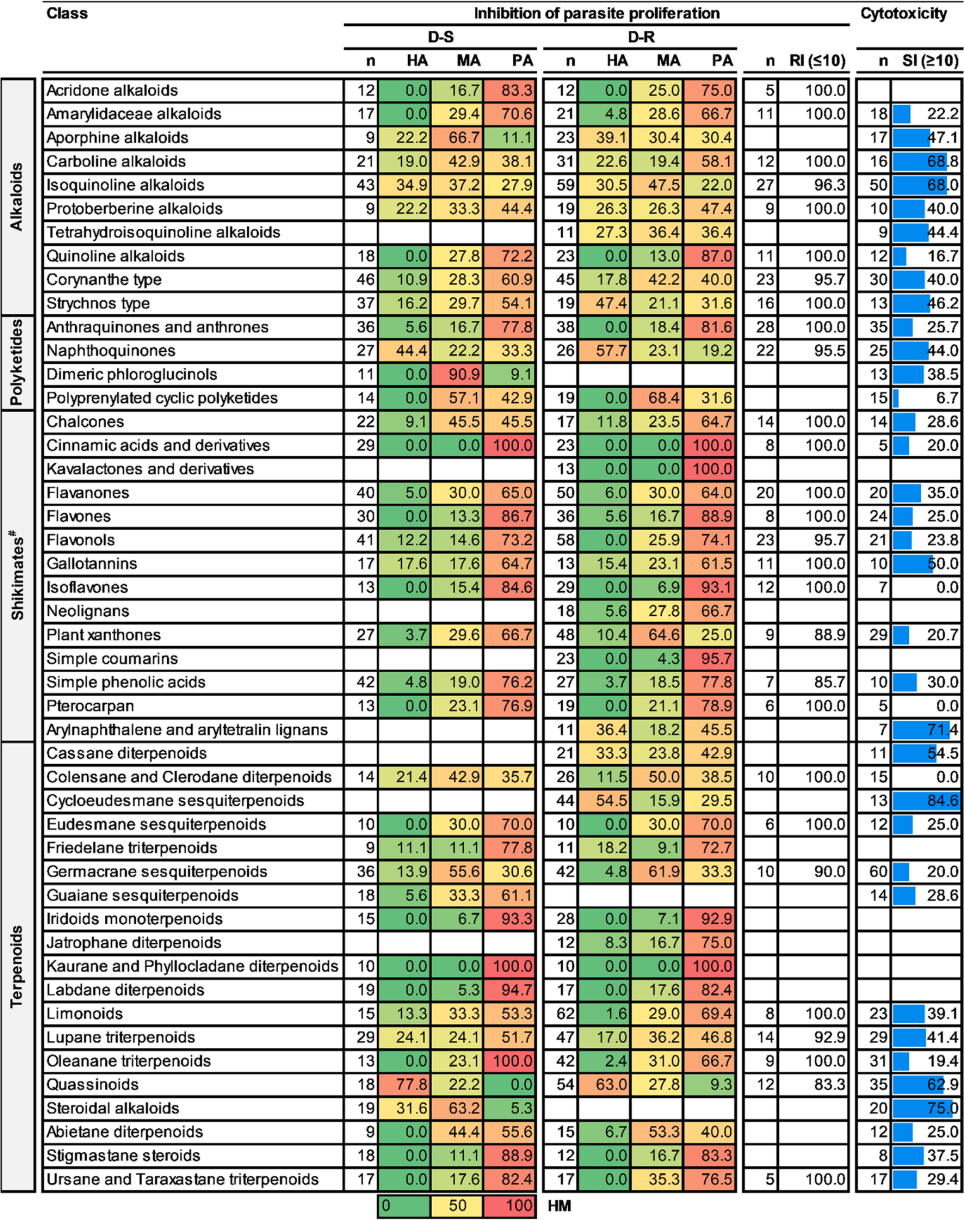
Antiplasmodial activity and cytotoxicity of higher plant-derived natural compound classes

n = number of compounds; HA, MA and PA values are expressed in % of total compounds evaluated; RI shows % number of compounds with RI ≤ 10; SI shows % number of compounds with SI ≥ 10. The heat map (HM) ranges from green (lowest value, 0%) to yellow (mid-range value, 50%) to red (highest value, 100%), visually illustrating the proportion of compounds classified as either HA, MA or PA. ^#^Shikimates and Phenylpropanoids

Overall, consistent with earlier observations, the alkaloid pathway and its associated sub-categories showed the most promising pharmacological properties including potency, RI and SI. Except for a few “pockets of brilliance”, e.g., quassinoids, cycloeudesmane sesquiterpenoids, and steroidal alkaloids, by and large, most compounds assigned to the terpenoid and shikimates and phenylpropanoid pathways and related super classes and classes were classified as PA.

### Drug-likeness assessment of compounds produced by different plant orders and families

Having assessed the pharmacological properties of plant-derived compounds assigned to different structural categories, we then investigated their drug-likeness for reasons outlined earlier (Moyo et al. submitted). Drug-likeness assesses the probability of a molecule to be bioavailable [18]. The current assessment was based on evaluating the in silico computed properties of compounds against criteria outlined by the Medicines for Malaria Venture (https://www.mmv.org/frontrunner-templates), Lipinski’s Rule of 5 [19], Veber’s rule [20] and Ghose filters [21]. To a large extent, most compounds in different pathways, super classes and classes complied with the set criteria. The most notable non-compliant structural classifications were the super classes fatty acids (rotatable bonds (RB) = 18) and steroids (molecular weight (MW) = 552, molecular refractory (MR) = 159), and the classes cycloeudesmane sesquiterpenoids (of super class triterpenoids) (MW = 599), kaurane and phyllocladane diterpenoids (of super class diterpenoids) (MW = 768, hydrogen bond acceptors (HBA) = 16, hydrogen bond donors (HBD) = 10, MR = 181, and total polar surface area (TPSA) = 272), and gallotannins (of super class Phenolic acids (C6-C1)) (MW = 628, HBA = 17, MR = 10, and TPSA = 302). All structural classifications showed an acceptable PAINS score (Moyo et al. submitted). However, some classes demonstrated marked synthesis accessibility scores (≥ 6.5), most notable kaurane and phyllocladane diterpenoids (8.7), jatrophane diterpenoids (of super class diterpenoids) (7.2), cycloeudesmane sesquiterpenoids (of super class triterpenoids) (7.3), stigmastane steroids (of super class steroids) (6.7), limonoids (of super class cyclic polyketides) (6.6), polyprenylated cyclic polyketides (Hop meroterpenoids) (of super class triterpenoids) (6.9), and oleanane triterpenoids (of super class triterpenoids) (6.9), see Tables 4, 5, 6.

**Table 4:**
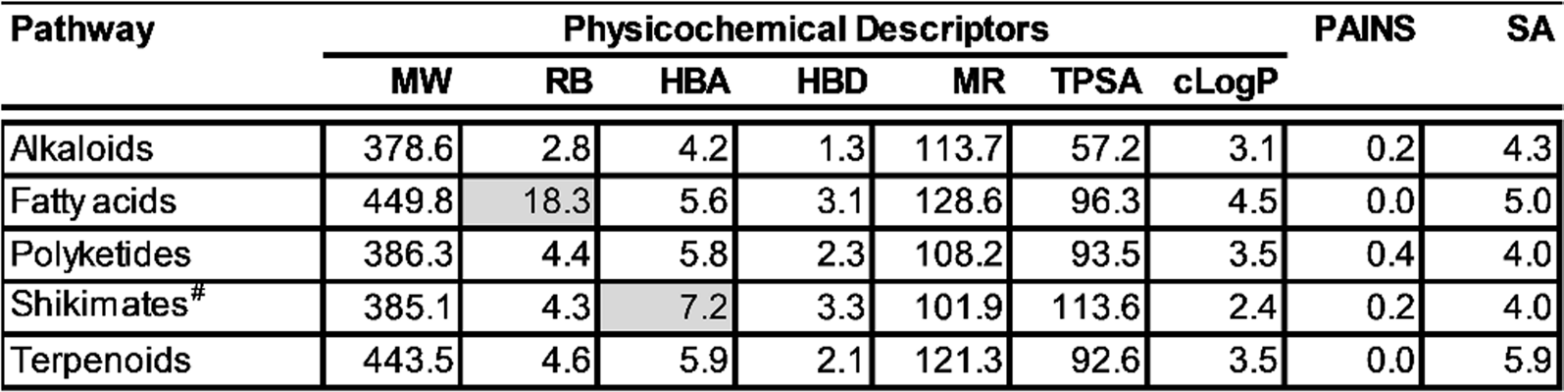
Calculated mean physicochemical descriptors for natural products in different pathways

*MW* molecular weight, *RB* rotatable bonds, *HBA* hydrogen bond acceptors, *HBD* hydrogen bond donors, *MR* molar refractivity, *TPSA* total polar surface area, *cLogP* consensus LogP, *PAINS* pan-assay interference compounds, *SA* synthesis accessibility

Grey shaded figures are those which don’t meet the set criteria; ^#^Shikimates and Phenylpropanoids

**Table 5:**
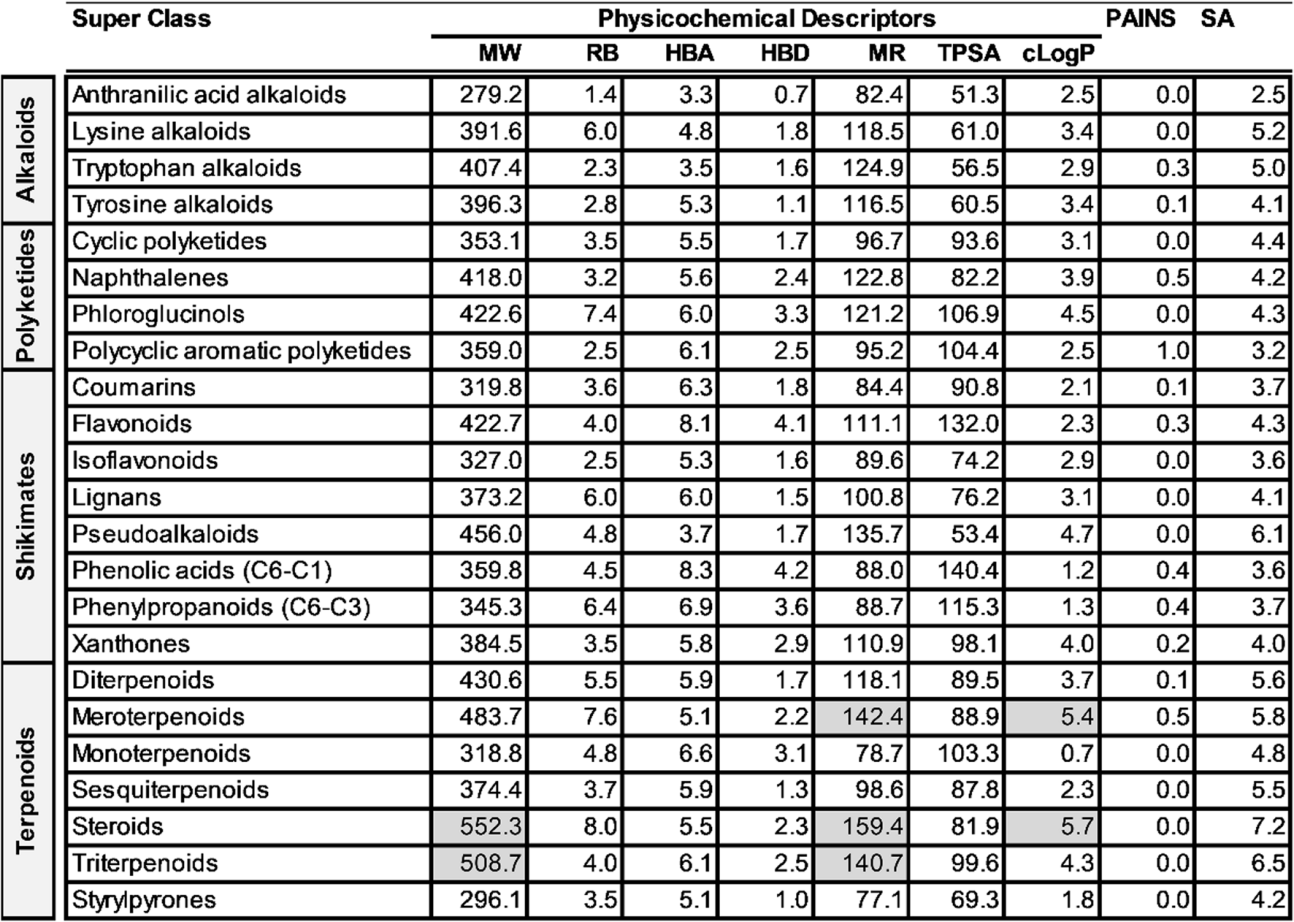
Calculated mean physicochemical descriptors for natural products in different super classes

*MW* molecular weight, *RB* rotatable bonds, *HBA* hydrogen bond acceptors, *HBD* hydrogen bond donors, *MR* molar refractivity, *TPSA* total polar surface area, *cLogP* consensus LogP, *PAINS* pan-assay interference compounds, *SA* synthesis accessibility

Grey shaded figures are those which don’t meet the set criteria; ^#^Shikimates and Phenylpropanoids

**Table 6:**
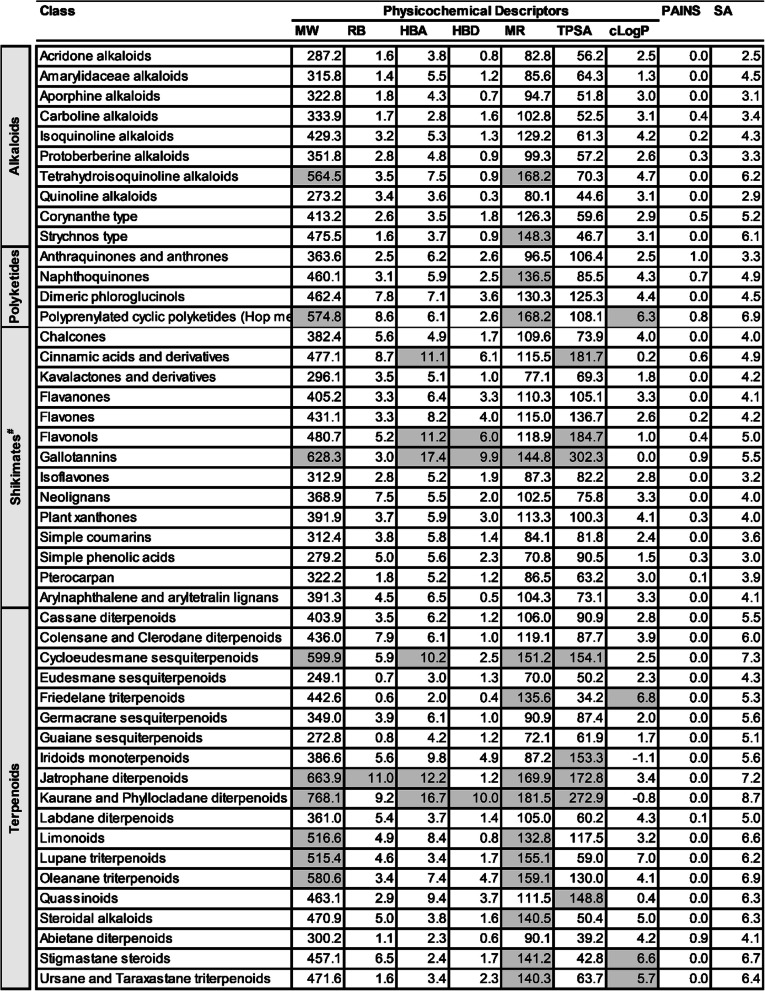
Calculated mean physicochemical descriptors for natural products in different classes

*MW* molecular weight, *RB* rotatable bonds, *HBA* hydrogen bond acceptors, *HBD* hydrogen bond donors, *MR* molar refractivity, *TPSA* total polar surface area, *cLogP* consensus LogP, *PAINS* pan-assay interference compounds, *SA* synthesis accessibility

Grey shaded figures are those which don’t meet the set criteria; ^#^Shikimates and Phenylpropanoids

### Overall ranking to identify compound structural classes for prioritisation in malaria drug discovery projects.

Having evaluated both pharmacological properties and drug-likeness we then employed an unbiased scoring system to rank different compound pathways, super classes and classes (Tables 7, 8, 9). For this process we decided against the inclusion of drug-likeness and RI as components as we noted that most compounds were compliant with these parameters.

**Table 7:**
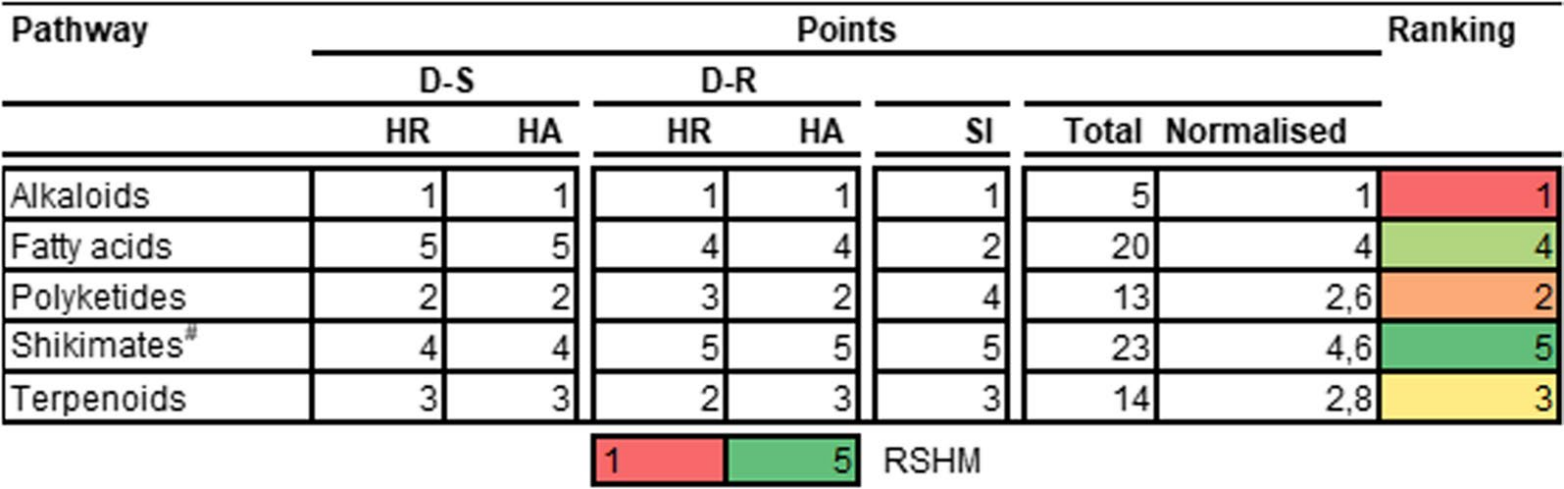
Ranking compound pathways for antimalarial drug discovery from higher plants

*HR* Hit rate; ranking score heat map (RSHM) ranges from red (best ranking, lowest points) to green (lowest ranking, most points). ^#^Shikimates and Phenylpropanoids

**Table 8:**
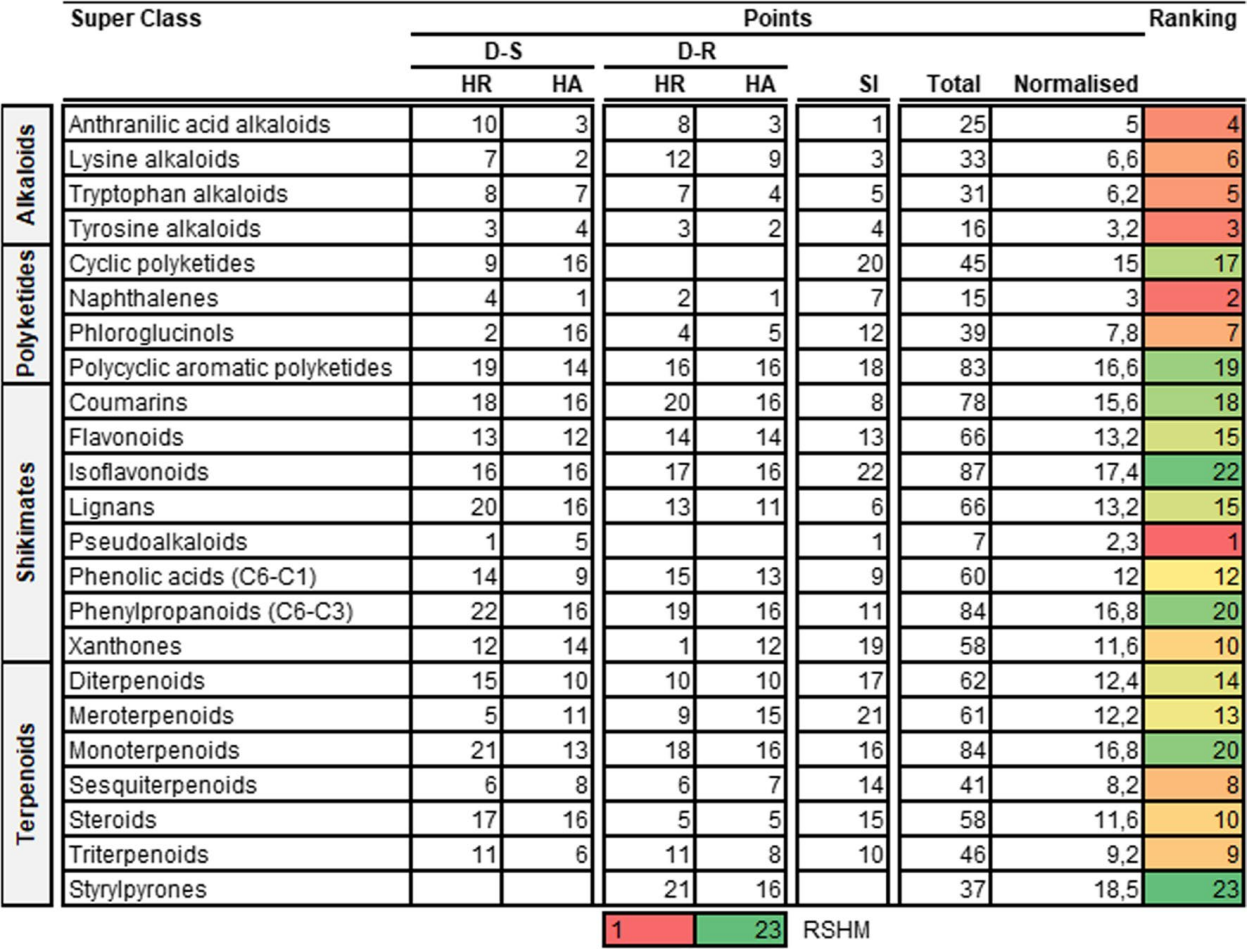
Ranking compound super classes for antimalarial drug discovery from higher plants

*HR* hit rate; ranking score heat map (RSHM) ranges from red (best ranking, lowest points) to green (lowest ranking, most points). ^#^Shikimates and Phenylpropanoids

**Table 9:**
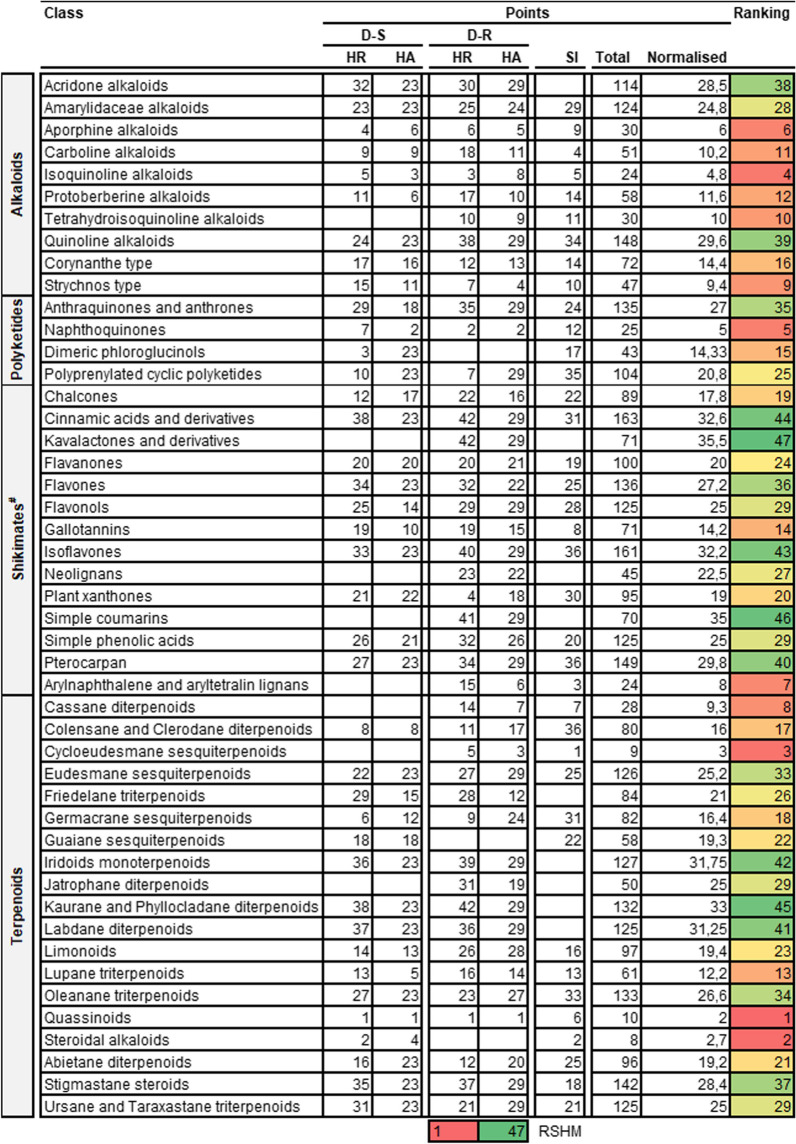
Ranking compound classes for antimalarial drug discovery from higher plants

*HR* hit rate; ranking score heat map (RSHM) ranges from red (best ranking, lowest points) to green (lowest ranking, most points). ^#^Shikimates and Phenylpropanoids

**Fig. 5:**
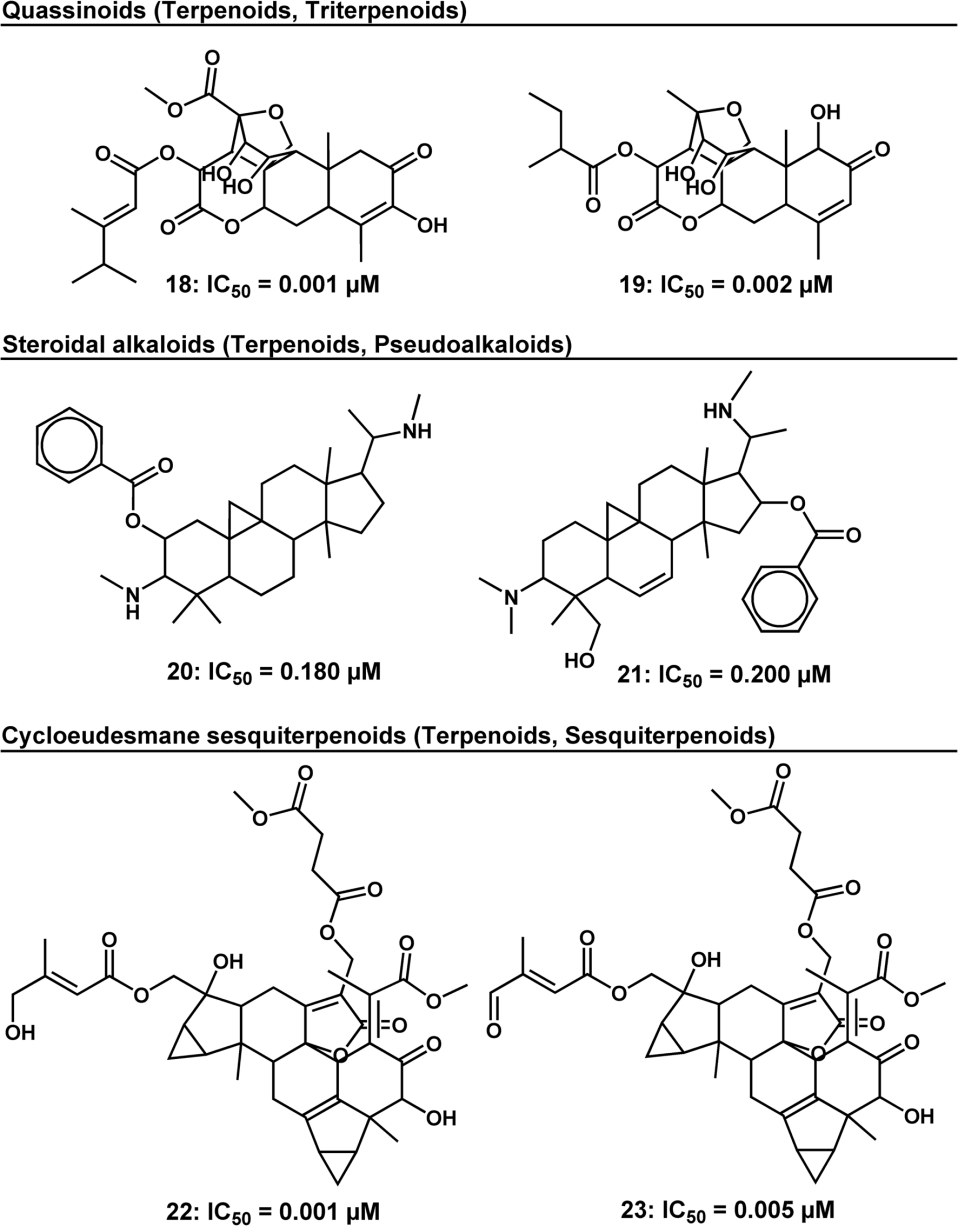

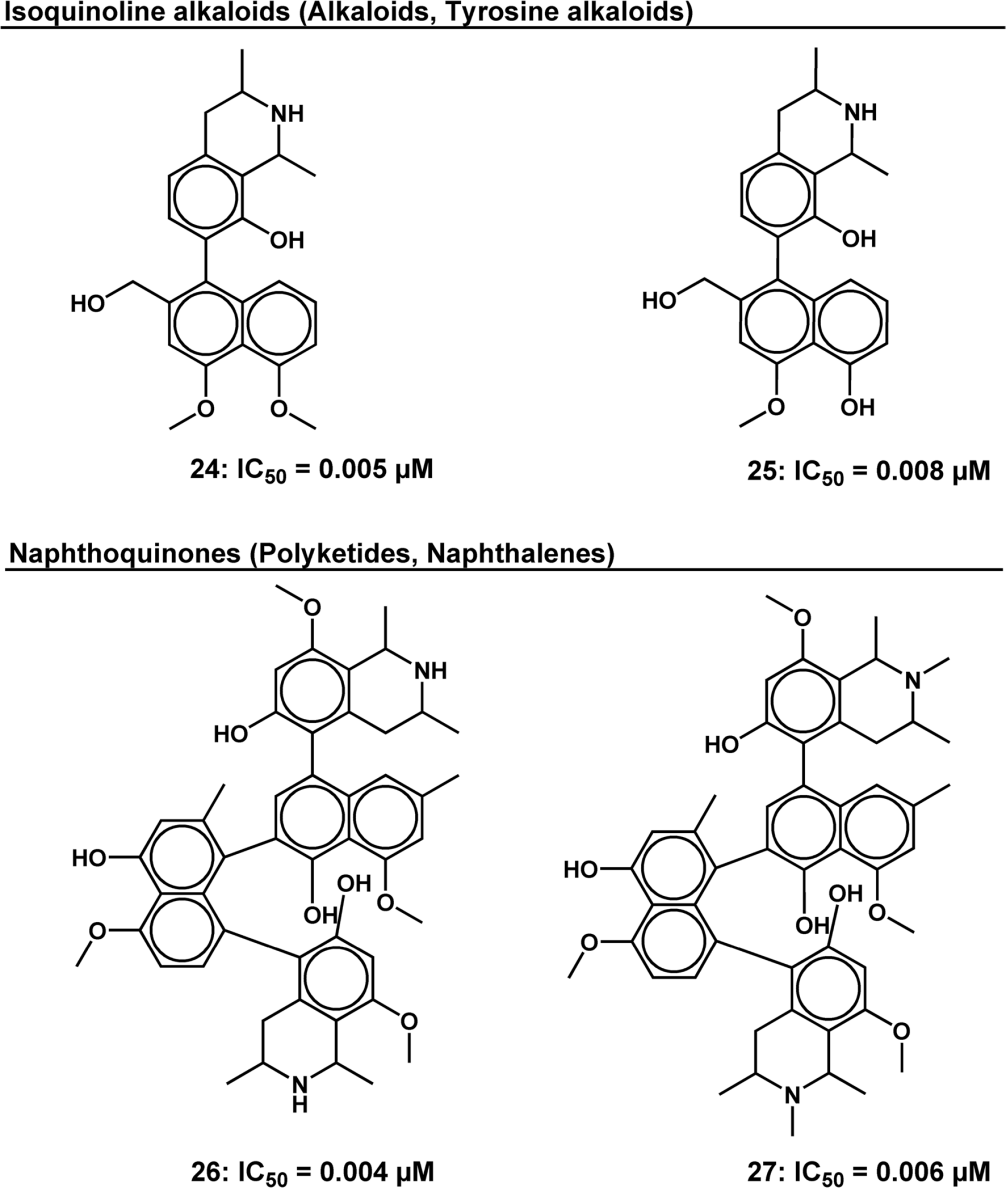
2D illustration of representative HA natural product compound structures from the top five ranked compound classes. Indicated IC_50_’s are measured against intra-erythrocytic asexual *P. falciparum* parasites

Following implementation of this scoring system, the following results were realized: the top-ranked pathway was the alkaloids, top three ranked super classes were (i) pseudoalkaloids, (ii) naphthalenes and (iii) tyrosine alkaloids and top five ranked classes were (i) quassinoids, (ii) steroidal alkaloids (iii) cycloeudesmane sesquiterpenoids (iv) isoquinoline alkaloids and (v) naphthoquinones (Fig. 5).

Structurally, quassinoids are degraded triterpenes which are highly oxygenated [22, 23]. They are subdivided into 5 types based on the carbon skeleton structure, i.e., C-18, C-19, C-20, C-22, and C-25. In nature, they are mainly confined to the Simaroubaceae family being responsible for the bitter taste of species in this family [22, 23]. They are complex molecules which are difficult to synthesise. Nonetheless, innovative synthesis approaches for this class of compounds have been reported in several studies [24–28] and concisely reviewed by others [29, 30]. Biologically, quassinoids have demonstrated exceptional potency across different disease areas including cancer, HIV and, as noted in this study, malaria [22, 23]. Some compounds have shown single digit nanomolar activity against both D-S and D-R *P. falciparum* parasites, not sharing cross resistance with chloroquine [31, 32]. It is therefore surprising that limited attention in the malaria field has been accorded this class of compounds. Almost half of the compounds analysed in this study were from two studies carried out by O’Neil and co-workers in 1986 and 1987 [31, 32]. One possible deterrent is the earlier discussed complexity in the synthesis of compounds in this quassinoids class. A work around for this could be to identify molecular features associated with potency and to use that to synthesise libraries of simple molecules whose structures are inspired by this class of compounds. This approach has gained impetus in the field with millions of virtual natural product-like compounds having been generated for drug discovery [33–35].

The steroidal alkaloid class of phytochemicals is limited in its distribution, amongst higher plants, being mainly confined to a few plant families that include the Solanaceae, Buxaceae, Apocynaceae, and Liliaceae sensu lato [36, 37]. Structurally, steroidal alkaloids consist of a basic steroidal skeleton containing either one or two nitrogen atoms either in the rings or on an attached functional group [37]. From a medicinal chemistry perspective, this complex class of compounds presents a synthesis challenge mainly brought about by the chiral nature of the molecules. This challenge was already noted by the high synthesis accessibility score highlighted in this study. Nonetheless, despite their complexity, several steroidal alkaloids have been successfully synthesised [38–40]. This has allowed for structural modifications leading to the development of analogues of parent compounds. Synthesis of analogues has resulted in the establishment of comprehensive SAR studies which have informed the progress of initial leads to clinical candidates [38–40].

The unique structural combination of the steroidal and alkaloid moieties endows this class of natural compounds with a unique set of physicochemical and biological properties. Consequently, they have a wide spectrum of biological activity including anti-inflammatory, antimicrobial and anticholinergic [36]. They have received marked interest in the cancer field for their exceptional activity epitomised by the clinically approved prostate cancer therapeutic abiraterone acetate [37]. Another noteworthy antineoplastic agent of the steroidal alkaloids class is cyclopamine, a natural compound isolated from the plant *Veratrum californicum* (Melanthiaceae) [37]. While cyclopamine is still in clinical studies, two of its analogues, vismodegib and sonidegib have already been approved by the Food and Drug Agency for cancer treatment [37]. Overall, steroidal alkaloids, despite their complex structure, are evidently amenable to structural modifications which have successfully led to the development of drugs.

Despite their exceptional biological activity, steroidal alkaloids have received comparatively subdued attention for their antiprotozoal activity, particularly against *P. falciparum* parasites. Foremost in their examination has been a study carried out by Szabo et al. [41] in which 25 alkaloids, including steroidal alkaloids (as per classification of NPClassifier) were isolated from *Buxus sempervirens* (Buxaceae). Their study provided evidence of the potential of this class of compounds, with five compounds showing exceptional activity with IC_50_ values < 1 µM [41]. Moreover, the authors highlight that SAR studies are in progress and are anticipated to give insights on structural modifications that could improve the potency of the compounds and reduce their toxicity, hence improving their selectivity further [41].

Cycloeudesmane sesquiterpenoids belong to the same super class category as artemisinin, namely the sesquiterpenoids. Despite their prolific activity, this class of compounds is yet to receive marked attention in the antimalarial drug discovery field. All compounds assessed in this study were from a single study by Zhou et al. [42]. To our best knowledge, there are few antimalarial studies that include this class of compounds, making it an interesting exploratory prospect. In contrast, the isoquinoline and naphthoquinone classes have received an abundance of antiplasmodial research attention [43–46]. These alkaloids have been isolated principally from species of African Ancistrocladaceae and Dioncophyllaceae, providing hope that they will be the source of the first plant-derived antimalarial drug to be discovered on the African continent.

**Fig. 6:**
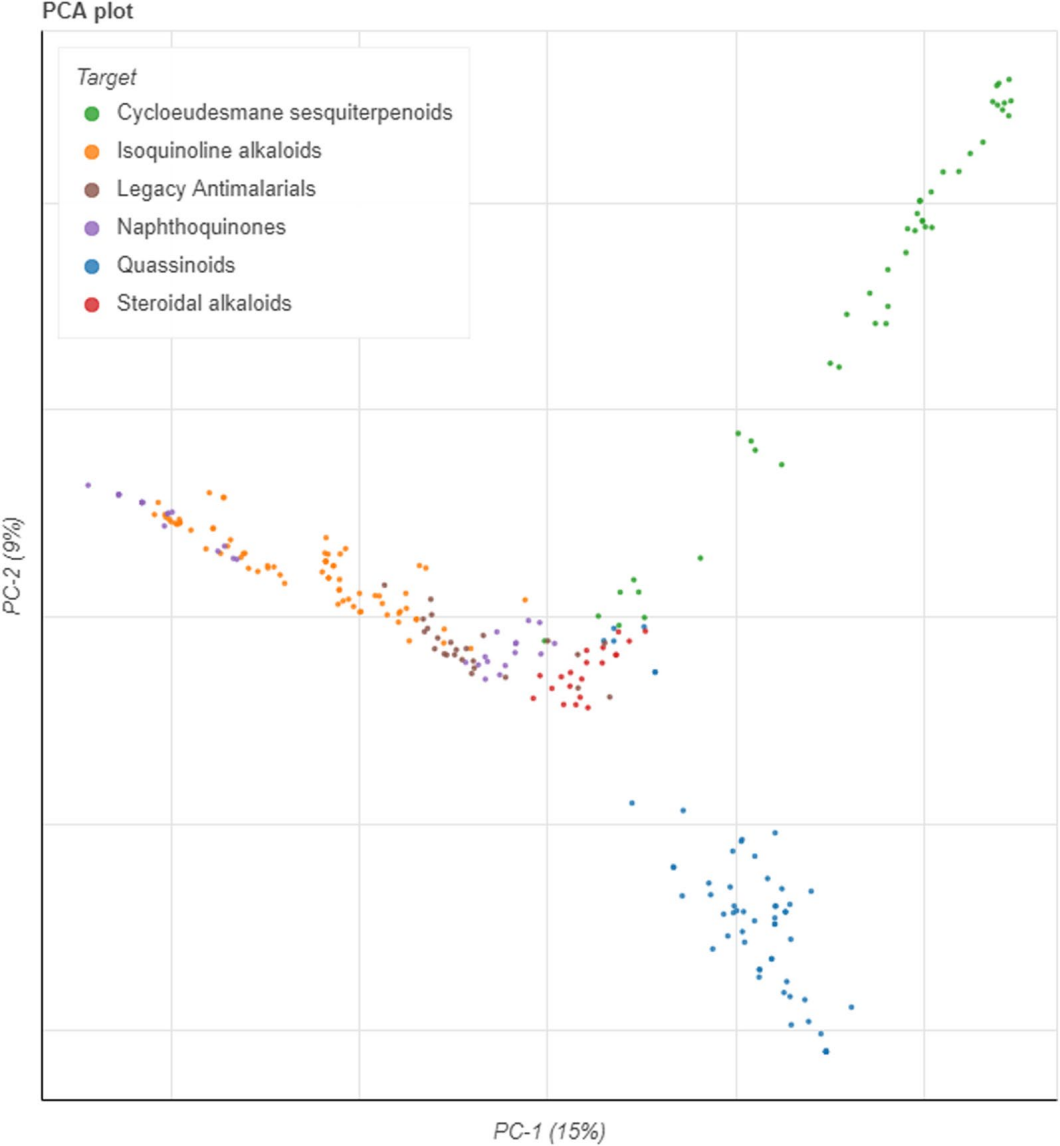
Launched drug chemical space (generated using the principal component analysis (PCA) dimensional reduction method) of the ‘legacy’ antimalarials and natural product compounds from the top five ranked compound classes. The online Python library for chemical space visualization, ChemPlot, was used to launch the chemical space of the natural compounds and ‘legacy’ antimalarials

**Fig. 7:**
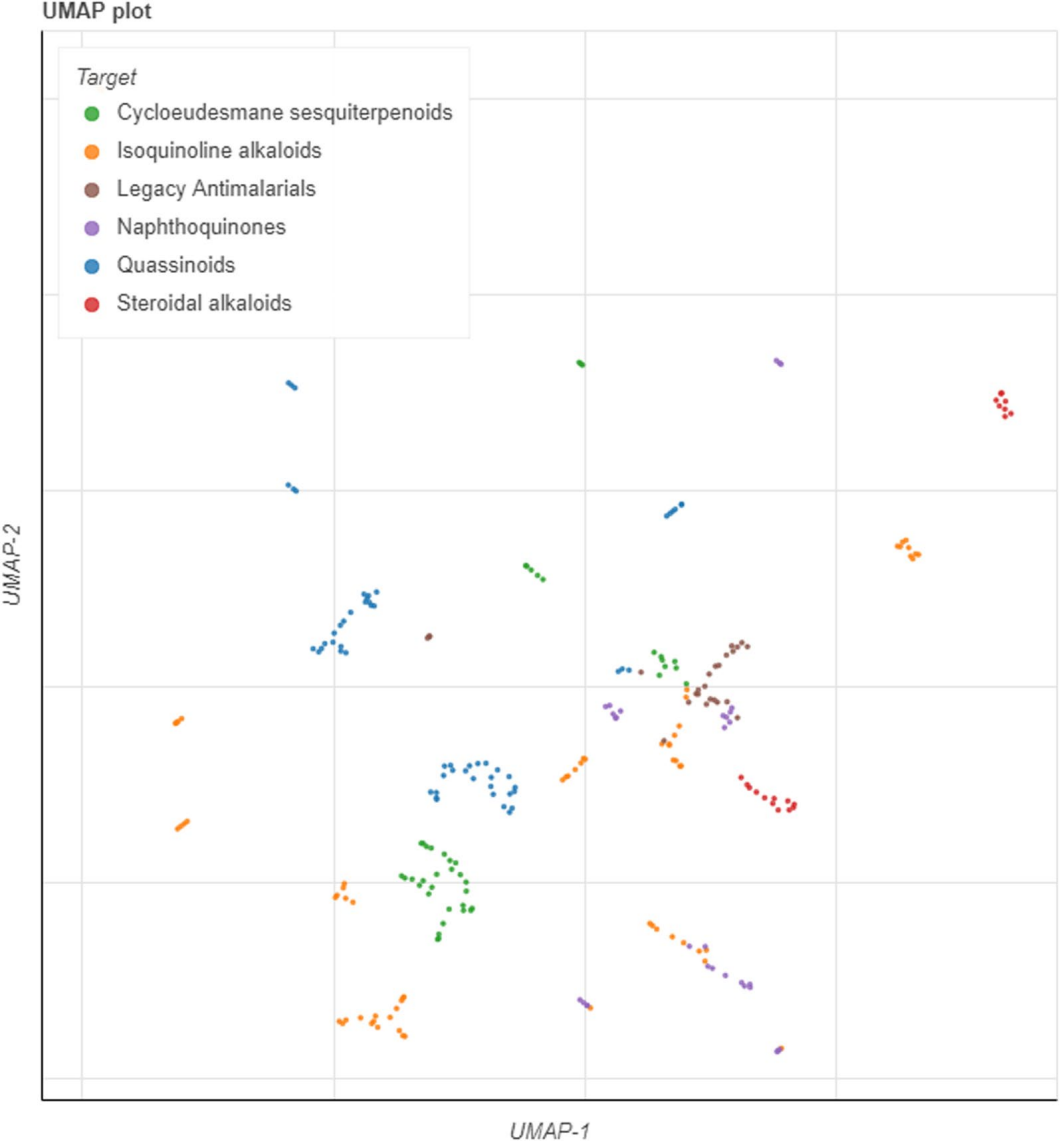
Launched drug chemical space (generated using the uniform manifold approximation and projection (UMAP) dimensional reduction method) of the ‘legacy’ antimalarials and natural product compounds from the top five ranked classes. The online Python library for chemical space visualization, ChemPlot, was used to launch the chemical space of the natural compounds and ‘legacy’ antimalarials

Following identification of the top-ranked classes of natural product compounds, we have sought to structurally compare them with the legacy set of antimalarials, i.e., the old and currently used malaria therapeutics [47]. To assess the structural similarity between the legacy antimalarial series and compounds assigned to the top five ranked classes, we launched the chemical space of the two groups of compounds (Figs. 6, 7). The chemical space of compounds is a multi-dimensional environment in which structurally similar molecules are grouped closely together [48] while structurally distinct compounds occupy a different space. The co-ordinates of the compounds within the 2D chemical space were plotted using four-dimensional reduction tools. In this study we chose two different dimensional reduction tools, namely the principal component analysis (PCA) and uniform manifold approximation and projection (UMAP) [48]. Encouragingly, compounds from the top five ranked classes largely clustered separately from the legacy antimalarials indicating structural differences between the two groups of compounds (Figs. 6, 7). Overlap in the chemical space was noted for the legacy antimalarials, and isoquinoline alkaloids in the PCA plot. This overlap could be explained by some structural similarities between the closely related isoquinoline alkaloids and quinoline derivatives which largely dominate the antimalarial legacy compounds. Nevertheless, most of the natural product classes occupied a different chemical space to legacy antimalarials, indicative of structural differences between these compound groups.

In conclusion, higher plant-derived natural products present a myriad of biologically exciting, structurally complex, and diverse molecules with great potential for development into antimalarial drugs. They offer a great avenue to find novel chemotypes that could circumvent the antimalarial drug resistance threat. Targeted isolation of compounds offers a great opportunity to isolate structurally related compounds of antiplasmodial interest that can be subjected to a fully-fledged drug discovery program, to expedite product delivery in a malaria drug discovery pipeline. Our study has provided insights to support this notion by cogently identifying structural classes of phytochemicals with acceptable in vitro antiplasmodial potency, RI, SI and drug-likeness properties. These compound classes (and super classes) should rationally be prioritized for target-based natural product drug discovery in the development of novel antimalarial chemotypes.

## Methods

Detailed methods for this article are provided in our companion article Moyo et al. (submitted).

## Data Availability

The data that support the findings of this study are available from the corresponding author upon reasonable request.

## References

[1] WHO, World malaria report 2022. 2022: World Health Organization.

[2] Menard D, Dondorp A (2017). Antimalarial drug resistance: a threat to malaria elimination. Cold Spring Harb Perspect Med.

[3] Takala-Harrison S (2015). Independent emergence of artemisinin resistance mutations among *Plasmodium falciparum* in Southeast Asia. J Infect Dis.

[4] Wells TNC (2011). Natural products as starting points for future anti-malarial therapies: going back to our roots?. Malar J.

[5] Gaillard T (2016). Antibiotics in malaria therapy: which antibiotics except tetracyclines and macrolides may be used against malaria?. Malar J.

[6] Organization WH. Guidelines for the treatment of malaria. 2015: World Health Organization. 26020088

[7] Spížek J, Řezanka T (2004). Lincomycin, clindamycin and their applications. Appl Microbiol Biotechnol.

[8] Nelson ML, Levy SB (2011). The history of the tetracyclines. Ann N Y Acad Sci.

[9] Peters W (1999). The evolution of tafenoquine—antimalarial for a new millennium?. J R Soc Med.

[10] Watson JA, Nekkab N, White M (2021). Tafenoquine for the prevention of *Plasmodium vivax* malaria relapse. Lancet Microbe.

[11] Wang M (2016). Sharing and community curation of mass spectrometry data with Global Natural Products Social Molecular Networking. Nat Biotechnol.

[12] Dührkop K (2019). SIRIUS 4: a rapid tool for turning tandem mass spectra into metabolite structure information. Nat Methods.

[13] Chassagne F (2019). The landscape of natural product diversity and their pharmacological relevance from a focus on the Dictionary of Natural Products^®^. Phytochem Rev.

[14] Egieyeh SA (2016). Prioritization of anti-malarial hits from nature: chemo-informatic profiling of natural products with in vitro antiplasmodial activities and currently registered anti-malarial drugs. Malar J.

[15] Kim HW (2021). NPClassifier: a deep neural network-based structural classification tool for natural products. J Nat Prod.

[16] Djoumbou Feunang Y (2016). ClassyFire: automated chemical classification with a comprehensive, computable taxonomy. J Cheminformat.

[17] Hai Y (2022). Trends of antimalarial marine natural products: progresses, challenges and opportunities. Nat Prod Rep.

[18] Daina A, Michielin O, Zoete V (2017). SwissADME: a free web tool to evaluate pharmacokinetics, drug-likeness and medicinal chemistry friendliness of small molecules. Sci Rep.

[19] Lipinski CA (1997). Experimental and computational approaches to estimate solubility and permeability in drug discovery and development settings. Adv Drug Deliv Rev.

[20] Veber DF (2002). Molecular properties that influence the oral bioavailability of drug candidates. J Med Chem.

[21] Ghose AK, Viswanadhan VN, Wendoloski JJ (1999). A knowledge-based approach in designing combinatorial or medicinal chemistry libraries for drug discovery. 1. A qualitative and quantitative characterization of known drug databases. J Comb Chem.

[22] Chakraborty D, Pal A (2013). Quassinoids: chemistry and novel detection techniques.

[23] Duan Z-K (2021). Quassinoids: phytochemistry and antitumor prospect. Phytochemistry.

[24] Kraus GA, Taschner MJ (1980). Model studies for the synthesis of quassinoids. 1. Construction of the BCE ring system. J Org Chem.

[25] Herscovici J (1993). Stereocontrolled routes to functionalized [1,8-bc]naphthopyran. A study on the total synthesis of quassinoids and tetrahydronaphthalene antibiotics. J Org Chem.

[26] Gross RS, Grieco PA, Collins JL (1990). Synthetic studies on quassinoids: total synthesis of (±)-chaparrinone. J Am Chem Soc.

[27] Ziegler FE (1986). Practical routes to two functionalized decalones for the synthesis of quassinoids. J Org Chem.

[28] Thomas WP, Pronin SV (2021). A concise enantioselective approach to quassinoids. J Am Chem Soc.

[29] Pazur EJ, Wipf P (2022). Recent syntheses and biological profiling of quassinoids. Org Biomol Chem.

[30] Kawada K, Kim M, Watt DS (1989). Synthesis of quassinoids. A review. Org Prep Proc Int.

[31] O'Neill MJ (1986). Plants as sources of antimalarial drugs: in vitro antimalarial activities of some quassinoids. Antimicrob Agents Chemother.

[32] O'Neill MJ (1987). Plants as sources of antimalarial drugs, Part 4: Activity of *Brucea javanica* fruits against chloroquine-resistant *Plasmodium falciparum* in vitro and against *Plasmodium berghei* in vivo. J Nat Prod.

[33] Tay DW (2023). 67 million natural product-like compound database generated via molecular language processing. Sci Data.

[34] Li Y (2018). Designing natural product-like virtual libraries using deep molecule generative models. Macromolecules.

[35] Yu MJ (2011). Natural product-like virtual libraries: recursive atom-based enumeration. J Chem Inf Model.

[36] Abd Karim HA, Ismail NH, Osman CP (2022). Steroidal alkaloids from the apocynaceae family: their isolation and biological activity. Nat Prod Commun.

[37] Xiang M-L (2022). Chemistry and bioactivities of natural steroidal alkaloids. Nat Prod Bioprospect.

[38] Sharpe RJ, Johnson JS (2015). A global and local desymmetrization approach to the synthesis of steroidal alkaloids: stereocontrolled total synthesis of paspaline. J Am Chem Soc.

[39] Tokuyama T, Daly J, Witkop B (1969). Structure of batrachotoxin, a steroidal alkaloid from the Colombian arrow poison frog, *Phyllobates aurotaenia*, and partial synthesis of batrachotoxin and its analogs and homologs. J Am Chem Soc.

[40] Zha X (2007). Efficient synthesis of solasodine, *O*-acetylsolasodine, and soladulcidine as anticancer steroidal alkaloids. Chem Biodivers.

[41] Szabó LU (2021). Antiprotozoal nor-triterpene alkaloids from Buxus sempervirens L. Antibiotics.

[42] Zhou B (2017). Nanomolar antimalarial agents against chloroquine-resistant *Plasmodium falciparum* from medicinal plants and their structure–activity relationships. J Nat Prod.

[43] Lombe BK, Feineis D, Bringmann G (2019). Dimeric naphthylisoquinoline alkaloids: polyketide-derived axially chiral bioactive quateraryls. Nat Prod Rep.

[44] Bringmann G (2013). Highly selective antiplasmodial naphthylisoquinoline alkaloids from *Ancistrocladus tectorius*. Phytochemistry.

[45] Bringmann G (2003). Habropetaline A, an antimalarial naphthylisoquinoline alkaloid from *Triphyophyllum peltatum*. Phytochemistry.

[46] Moyo P (2020). Naphthylisoquinoline alkaloids, validated as hit multistage antiplasmodial natural products. Int J Parasitol Drugs Drug Resist.

[47] Charman SA (2020). An in vitro toolbox to accelerate anti-malarial drug discovery and development. Malar J.

[48] Cihan Sorkun M (2022). ChemPlot, a Python library for chemical space visualization. Chem Methods.

